# Compulsory Health Insurance

**Published:** 1920-09

**Authors:** Jerry McQuade


### COMPULSORY HEALTH INSURANCE

ALARMED BY REPORT THAT WEALTHY INTERESTS INTEND FORCING LEGISLATION ALONG THIS LINE, NEW YORK AND NEW JERSEY PHYSICIANS, DENTISTS AND DRUGGISTS ORGANIZE PROFESSIONAL GUILD TO WAGE DEFENSIVE FIGHT—BITTER FIGHT AHEAD.

*Back of the Health Insurance legislation, which is to be introduced for enactment into law, into all state legislative bodies of the country, in the next three months, is said to be powerful influence, money and prestige. This statement, made by two speakers at a mass meeting of physicians, dentists and druggists called December 4th in the New York College of Pharmacy, for the purpose of organizing a professional guild to combat this class of legislation, reacted like a bombshell filled with T. N. T. on the three professions represented at the conference.*

With powerful interests supplying the brains, the funds and the propelling force to push and force through this paternalistic legislation, which will make heavy inroads into the revenues of all three professions, speakers addressing the meeting, declared it was a life and death struggle, to defeat which the professions must unite and utilize every agency at their disposal as a combative force.

Unless this is done, the speakers stated, the interests favoring the legislation would win and the physician, dentist and druggist lose his individual standing in the community to become part of a machine, with a Damocletian sword hanging constantly over his head.

As a corollary of the Health Insurance Bill, Dr. J. A. O'Reilly, chairman of the Professional Guild of Kings County, New York—one of the first of the professional guilds of physicians, dentists and druggists organized in New York state—told the conference the backers of Health

Insurance intend to introduce a Medical Practices Act, requiring physicians to register with the state each year.

"If this act becomes a law," said Dr. O'Reilly, "any physician who refuses to join the Health Insurance panel and agree to give his professional service to employes insured under the act at the nominal charge fixed by the panel, would be in danger of losing his license."

"The physician's license to practice medicine," declared Dr. O'Reilly, "is a discretionary privilege granted by the state, and is not an inherent, inalienable property right of the physician—what the state gives, it may take away."

Refusal to tender his service to the Health Insurance panel, the Doctor believes, would inevitably lead to the state's refusal in turn to renew the license of the offending physician when he applied for re-registration.

Proof that this is the plan which the designers of the Medical Practices Act have in mind, was supplied him recently, said Dr. O'Reilly, when State Senator Loring Brown informed him that any physician, dentist or druggist who refused to make the Health Insurance law operative, when enacted, would have his license to practice his profession in the state taken away from him.

"In other words," explained the Doctor, "we are to be destroyed unless our three professions supinely surrender our manhood and our right to the living to which we have consecrated our lives. While it may be true that our right to a license is alienable and that the state, out of pique, may take it away from us, the fact remains that our economic right is the same as that of any other citizen. Economically the right of the physician, dentist and druggist is no different from that of the longshoreman. If you cut down the wages of the longshoreman, he has the right to protect himself—our right is precisely the same.

"The Davenport-Donahue Bill, which the proponents of Compulsory Health Insurance sponsor, generously provides that we may appeal, if the fee fixed by the panel is, in our judgment, unfair. This is a joke. To make an appeal, we must print the record of the case, the cost of which would be many times the maximum sum we might claim for our service. In other words, the very thought of an appeal is prohibitory, and we must accept what is allotted us or leave it—we are the victims and only God can help us.

"As soon as this law is enacted, some political doctor, who in skill, technique and all-round knowledge would not be fit to wash the instruments of a real doctor, will be placed in charge of the district panel. This political doctor at the head of the panel, will fix the fees of every practitioner in his neighborhood in every insurance case in which he may be called to give his service; also the fees of the dentist and druggist. *The incompetents will thus pass on the competents.*

"Of course the politicians favor the plan, because it makes available thousands of new jobs for their satellites, at *our expense.* The state may do what it wants to do with its own money, but it has no moral right to be generous with our money, and that is exactly what it proposes by this legislation.

"Today the statistics show that out of every dollar a man earns, seven and one-half cents is the cost of maintaining human health; we maintain that the state has no right to say that in future, through the operation of a health insurance act, it must have this same service for two and one-half cents, any more than it has a right to say to other men that in future they must sell for thirty-three cents what they now sell for one dollar. Back of this whole bill is money, a false altruism, conceived in Germany and imported as a rich man's sop to the forces of unrest."

Dr. William T. Anderson, dean of the Brooklyn College of Pharmacy, followed and urged that New York county and other cities throughout the country immediately organize a professional guild of physicians, dentists and druggists, similar to the Kings county guild.

The Kings county guild now combines four thousand physicians, dentists and druggists, and has the endorsement of twenty professional and scientific societies representing the three professions in that county.

During the last campaign, the guild appointed working committees in each assembly district, composed of two physicians, two druggists and one dentist. This committee called on each candidate for the state assembly and state senate to ascertain his views on health insurance and ask for a pledge to vote against any bill providing for compulsory health insurance. Against those who expressed their intention to vote for such legislation, the guild waged a bitter fight, carried on under what was probably the most unique conditions under which a political campaign in this country has ever been waged.

Supplementing the regulation stump-speaking practice, characteristic of all campaigns, the professions carried the fight into the very sickroom. The doctor over the sick-bed of his patient, in the confidential relation of health minister in the home; the dentist working over his patient in the chair, and the druggist talking to his customers in his store, urged the defeat of those who admitted their intention to support health insurance.

Under this silent, overpowering, soft-pedal professional propaganda, ten hostile candidates went down to defeat and ten candidates pledged to oppose health insurance were elected in their place.

In the political history of America, no parallel for this novel campaign and demonstration of the professional man's power and influence with his patient, when put to the test, has ever been so conclusively established.

What has been done once can be done again. By capitalizing his strong personal influence with his patients and customers in this way, speakers stated that the physician, dentist and druggist have the opportunity to weld into a concrete force the greatest power possessed by any body of men in any community and can defeat the legislation proposed.

With women now a voting factor in each community, the doctor and the dentist have a powerful ally in every home. The doctor is the woman's friend, counselor and helper in trouble. He has brought her babies into the world, has protected and guarded them for her in sickness and been the saving grace between life and death for all those she loves—a vote to please and help him is asking little—of course he can have it. With the vote of the mother of the house—in many instances—he can have the vote of the man, too.

Those present at the meeting agreed to use this influence, and following a number of stirring addresses by other physicians, dentists and druggists, a guild was formally organized for New York county, with physicians as chairman and vice-chairman, a dentist as treasurer and a druggist as secretary. Announcement was made that similar professional guilds had been organized in other cities of the state and that a preliminary meeting attended by Dr. Dickenson, president of the New Jersey State Medical Association, had been held with a view to similar organizations in New Jersey.

As part of the plan to enlist the support of their patients in the effort to bring pressure to bear upon legislators favorable to compulsory health insurance, the Professional Guild of Kings County has prepared a letter which its physician members are to send to all of their patients which is most unusual in character. With this letter will be enclosed a letter which the patient is requested to sign, with the names of all members of her family who approve of it.

From New York and New Jersey the plan for a nation-wide establishment of professional guilds will be extended until every state in the union is represented. *Here we have a brand-new force in American politics.*

With the opening of the state legislatures this month the fight starts. To date, the professions have defeated this legislation for three years. For the fourth time the wealthy interests seeking its enactment are now ready to come forward again, this time with increased strength, more money and more powerful agencies than ever behind it.

Free doctors, free medicines, free maternity benefits and free funerals look good to the proletariat, and such legislation it is believed will help solve its state of unrest. It is Bismarck's old formula to keep the socialists from getting too belligerent and disturbing the crown, revamped to meet American conditions.

The fact that employers of labor must pay the bills, as an additional tax, beyond all other taxes they now have to pay—with which all this free stuff will be provided—counts nothing. *It's a bone for the restless and they must have it.*

If you have to help pay the bill in medicines and surgical supplies sold at approximate cost—that's your fault. If you want to escape it—*change your business.* If you want to stay in the business, this is the time to fight for it or forever keep your peace.—Jerry McQuade, in *Drug Topics.*

